# Harmonic organisation conveys both universal and culture-specific cues for emotional expression in music

**DOI:** 10.1371/journal.pone.0244964

**Published:** 2021-01-13

**Authors:** George Athanasopoulos, Tuomas Eerola, Imre Lahdelma, Maximos Kaliakatsos-Papakostas

**Affiliations:** 1 Dept of Music, Durham University, Durham, United Kingdom; 2 School of Music Studies, Aristotle University of Thessaloniki, Thessaloniki, Greece; Universiteit van Amsterdam, NETHERLANDS

## Abstract

Previous research conducted on the cross-cultural perception of music and its emotional content has established that emotions can be communicated across cultures at least on a rudimentary level. Here, we report a cross-cultural study with participants originating from two tribes in northwest Pakistan (Khow and Kalash) and the United Kingdom, with both groups being naïve to the music of the other respective culture. We explored how participants assessed emotional connotations of various Western and non-Western harmonisation styles, and whether cultural familiarity with a harmonic idiom such as major and minor mode would consistently relate to emotion communication. The results indicate that Western concepts of harmony are not relevant for participants unexposed to Western music when other emotional cues (tempo, pitch height, articulation, timbre) are kept relatively constant. At the same time, harmonic style alone has the ability to colour the emotional expression in music if it taps the appropriate cultural connotations. The preference for one harmonisation style over another, including the major-happy/minor-sad distinction, is influenced by culture. Finally, our findings suggest that although differences emerge across different harmonisation styles, acoustic roughness influences the expression of emotion in similar ways across cultures; preference for consonance however seems to be dependent on cultural familiarity.

## 1 Introduction

Music is prevalent in all cultures [1]. Its main relevance comes from a strong potential to communicate and induce emotions in listeners and participants of musical activities [2], as well as provide social cohesion in groups (both points made by [3]). Cross-cultural research on music and emotions have established that many of the emotions—whether these are affective dimensions [4], a few basic emotions [5], or complex emotions [6]—can be communicated across cultures at least on a rudimentary level of recognition. Listeners familiar with a specific musical culture have a clear advantage over those unfamiliar with it in emotion recognition tasks [6]. Nevertheless, the broad picture is that a fair amount of studies have highlighted success in emotion recognition in music across cultures. It is, however, less clear how listeners actually achieve this feat, particularly when very few studies have undertaken a manipulation of the cues for emotion recognition [1, 7], and none have done so by addressing harmonisation. Instead, there are suggestions of core cues (psychophysical) such as loudness, tempo, and acoustic roughness versus cultural cues such as melodic motives or harmonic organisation [4, 8, 9].

On the topic of cross-cultural research in music perception, there is work indicating that there may be innate properties underlying specific musical behaviours, based on the concept of human ethology [10]. In-depth research on whether universals in human psychology can inform whether innate properties exist as underlying musical behaviour has caught the attention of researchers in the last two decades. Earlier studies by Harwood [11] and further elaborated and categorized by Brown and Jordania [12] have indicated that perceptual universals exist across several structural elements in music, such as distinct pitch perception, perception of octaves, and melodic contour, among others. In addition to universal elements in the perception of structural components of music, significant work has also been done on the communication of emotional expression in music as a cross-cultural phenomenon [4–7, 13]. That being said, the concept of what constitutes humanly organised sound as intentional production of music itself is not universally agreed upon. There are cases, as reported by Blacking [14] where music by one culture may be dismissed as noise by another culture simply because the means of delivery is through a radio; Trehub et al. [15] have also reported that individuals from even the same cultural group may classify sounds differently from each other. Jacoby et al. [16] give further examples, such as the Muslim call to prayer and the use of sound as curative practice among Gnawa sufis in Morocco which by Western standards are often misunderstood as music due to their high degree of aestheticisation and the use of maqam tonal structures.

Western music’s harmonic organisation is often seen as one of the major differences between Western and much non-Western music [17]. In the Western musical culture harmonisation has developed over several centuries to create patterns of tension and release using particular building blocks (functional tonality, chords), syntax (sequencing these blocks) which have become conventions. In non-Western music, however, harmonic organisation as a concept manifests itself in a different way, using different building blocks or utilising different variations of voicings sounding together. These include, but are not limited to, heterophony between a leading voice and accompanying instruments, or for more contemporary non-Western music styles, via melody-dominated homophony. The perception of simultaneous sounds is closely related to the question of consonance and dissonance, which is a notoriously contentious topic in cross-cultural research on music perception. Despite the suggestion that humans might have a biological predisposition to prefer consonance over dissonance [18], cross-cultural research on the issue has not yet been able to settle this question: previous studies show both similarities [19] and differences [20] across cultures, as well as a complete lack of the perception of consonance outside the Western musical culture [21]. The most recent of these studies by McDermott et al. [21] has been criticised for its methodological confounds [22] which hinders the findings’ generalisability. However, previous research has demonstrated that familiarity on both on an individual and on a cultural level does have an impact on the perception of consonance and dissonance [23, 24], implying that its perception may vary across cultures due to familiarity.

Mode has been acknowledged as a solid cue for emotional expression in Western music since the pioneering days of Hevner [25] who established that music played in the major mode typically leads to happy and graceful expression, whereas music played in the minor mode to sad and dignified. More recent research has added little to this distinction [26–29], except to show that although young children can potentially make the distinction between the major mode and the minor mode and what these may signify in terms of emotion [30], it is a cue they need to learn before they are able to harness it in emotion recognition [31]. With regard to the question of how universal the affective distinction between major/minor is when taken outside the Western context, a recent study [32] found that Chinese listeners do make the Western-style affective distinction, that is, the major mode was linked to positive and high arousal, whereas minor was associated with negative and low arousal, although this was influenced by previous exposure to Western music. When non-Western participants who have no exposure to Western music—such as the Mafa tribe members in Cameroon [5]—provide answers to emotion recognition task using music in both major and minor mode, the results suggest that the mode of the piece is linked to the recognition. Both Westerners and Mafas classified the majority of major pieces as happy, the majority of pieces with indefinite mode as sad, and most of the pieces in minor as scared/fearful. However, this finding cannot be attributed to mode alone, particularly when other cues in music (tempo, timbre, note density, etc.) co-varied with mode. There is no evidence that Mafa music is organised according to major/minor mode and the recognition and the awareness for these pitch structural differences was not verified.

We presume that the major-minor distinction and its association with specific emotions is a Western cultural convention, and that it will have limited utility in communicating emotions in a cross-cultural setting if no additional cues are offered. Historically, the major-happy/minor-sad association in Western music dates back to the 14th century and its origins are disputed [33]. It has been suggested that in Western music the major mode is perceived as a standard form as opposed to the minor mode because music in major keys is more common, and that this is in turn would be due to the major triad’s closer similarity to the harmonic series [34, 35]. Two theoretical ideas have been put forward for why the minor mode in particular might lead to emotional expression consistent with sadness. The first theory relates to the properties of speech that link minor mode and sadness [36]. The second theory is relevant to the structure of melodies, in which the minor mode lends itself to the use of smaller intervals than the major mode, as small intervals are characteristic of sadness in speech [37]. Despite these two theoretical notions, which are backed up by some empirical observations, the direct evidence of the utility of mode in emotional expression in non-Western music has not been collected even if observations about mode as a universal cue in emotion recognition have been made [5].

Jacoby and his colleagues [16] have raised several valid points for cross-cultural research venturing into the domain of the psychology of music. Apart from highlighting the sampling bias in psychology towards recruiting Western educated participants originating from industrialised rich democracies, the authors stress the importance of conducting cross-cultural research in music by first assessing how music is viewed and how it functions among participating cultures, something which ought to inform the way that the research questions are put together. This would deem any results obtained from said research to be both comparable and meaningful. Second, the authors bring to the attention of the reader the “tradeoff between experimental control and ecological validity” in cross cultural research, and ways to enhance both; this could be done either by using music samples and paradigms from the performance cultures under investigation, or by using extremely simple stimuli [38], or by trying to find common elements between diverse groups and put them to use [39]. Keeping these comments in mind, we acknowledge that cross-cultural research aiming to reveal how music harmonisation interacts with the emotional perception of music would require meeting the following points: 1) locating participants who are largely naïve to Western music; this, due to the overarching effect of globalization and the effect of Western musical styles on world culture [40] is easier said than done, but not impossible overall, 2) locating participants whose own music has established native performance traditions, for whom there is a specific focus on the expression of emotions through and with music, 3) locating participants whose music contains elements which have a concept of either melody-dominated homophony, heterophony, or harmony, so as not to be at a significant disadvantage to their Western counterparts in this key concept, 4) developing a suitable research methodology that would enable participants from different cultures to assess emotion expression in both Western and non-Western music in a meaningful manner that they comprehend, and 5) travel to the location of non-Western participants to collect data, in order to assess and ensure that only non-Western participants with low-level exposure to Western culture are enlisted. This is considered essential due to the fact that musical structural knowledge can be implicitly acquired through mere exposure to a particular type of music [41, 42].

### 1.1 Rationale and hypotheses

Our aim is to explore how participants largely unfamiliar with Western music styles will assess emotional connotations of various Western and non-Western harmonisation styles, and whether cultural familiarity with harmonic idiom such as the major/minor mode will consistently relate to emotion communication. Thus we focus only on one musical feature, harmony, and keep others (tempo, timbre, register, melody) constant. We hypothesise three corollaries emerging, with the aforementioned conditions in place:

1. Western concepts of harmony are not as such relevant for participants unexposed to Western music when other emotional cues are kept constant (culture-specificity). This should hold true also for the major/minor mode and its association with negatively and positively valenced emotional expressions despite past observations [5].
2. When music utilises a familiar harmonic idiom, the harmonies alone are able to colour the emotional expression of music in the culturally appropriate manner.
3. The acoustic roughness of the harmonisation styles will have an impact on the emotional expression (universal).

The last hypothesis relates varying levels of acoustic roughness created by different harmonisation styles. As early as 1885 von Helmholtz [35] proposed acoustic roughness as an explanation for why some pitch combinations are considered dissonant, tense and disagreeable. There is consensus that interference patterns between wave components of similar frequency gives rise to beating [43, 44], which in turn creates the sound quality of roughness that listeners typically perceive as unpleasant. The perception of roughness has a biological substrate, as beating occurs at the level of the basilar membrane in the inner ear when the frequency components are too near to separate [45]. Roughness has been linked to perceived anger in speech [46] and is used in both natural and artificial alarm signals as it confers a behavioural advantage to react rapidly and efficiently [47]. The sensitivity to roughness seems to be present cross-culturally [21], but its musical appraisal differs significantly across musical styles and cultures: although a typical Western listener perceives roughness as disagreeable and negative in valence [48], it is deliberately harnessed in the vocal practice of “beat diaphony” (known as “Schwebungsdiaphonie” in German literature) in the Baltic and Balkan regions of Europe [49] and in Papua New Guinea [50]. All in all, the effect of roughness is seen as prevalent in dissonant, but not in consonant pitch combinations [51, 52]. For this reason we hypothesise that roughness will be a significant predictor of emotions in the different harmonisation styles that exhibit systematic variations in roughness [46].

Taking into consideration our research hypotheses and rationale, the most appropriate fieldwork location found by the authors to put these hypotheses to the test was the region of Khyber Pahtunkwa in remote northwest Pakistan. Two ethnic groups were located who matched the design demands: the Kalash, who are a remote Indo-European/Aryan polytheistic community, and the Chitrali/Kho people (note that the tribe is referred to as Khow, and the people are referred to as Kho) who are the native Muslim population of the Chitral region. Due to geographical, as well as cultural isolation and a lack of readily available technological amenities (internet, radio stations, access to world music, and a stable electricity grid), the two tribes match the description of Fang’s [32] Chinese listeners as truly “un-Westernised”.

In addition to the points above, we consider that it is necessary to mention that, although diversity between cultural groups is certainly very interesting as a research pursuit, the fact of the matter is that internal diversity within each group should also be taken into account, as cases of “uniqueness” presented in ethnographic reports may be due to a lack of thorough reporting and cross-comparison and examination of other cultural groups and societies in the region, or further afield on a global scale. Research conducted by [1, 53] clearly outlines that the existing variations found across cultural groups on their musical practices and behaviours are comparable, and often smaller than the variations found within each single cultural group. Though undoubtedly the richness of all the groups are worthy of thorough in-depth examination (and we present significant information about the tribes’ own musical practices in S4 Appendix so as to place them into proper context), we will simultaneously portray both between and within cultural group variation in the Results and Discussion section, and assess whether a similar pattern of higher within cultural variation in comparison to the variation between cultures will also be observed in this study.

The stimuli, the self-report measures and the validation operations are described in detail in the Supporting Information sections. Full examples of the scores and audio are available from Open Science Framework, https://osf.io/wq4tp/. In short, participants assessed the emotions in two melodies without any accompaniment which were also presented with eight different harmonisation styles: Bach Chorale, Jazz, Organum, and Whole-tone as well as Traditional Greek Epirote and one of the Kalash traditional styles (Drasailak) used in rituals. In addition, Bach and Jazz harmonisation styles were presented both in major and minor mode (same harmonisation but the third and sixth scale degrees were altered by a semitone). The participants consisted of two non-Western groups from the northwest Pakistan region of Khyber Pahtunkwa (Kalash and Kho), and Western participants from the United Kingdom (ethnic British monolinguals, hereafter as UK participants). We verified that the two pictorial self-report measures of emotional expression, which included an adaptation of Self-Assessment Mannequin (SAM) images [54] as well as facial expressions representing basic emotions taken from the Montreal Set of Facial Displays of Emotion (MSFDE) database [55] were clearly understood by the participants by initialising the experiment with a detailed session of attributing emotional speech and music expressions (see S1 Appendix).

## 2 Materials and methods

### 2.1 Procedure

Ethics approval was obtained from the host institution (MUS-2019-01-28T14:54:07 -kxhw42). The research project and its method were further assessed by the High Commission of Pakistan in London, and by the local authorities in Pakistan (Government of Khyber Pahtunkhwa, Home & Tribal Affairs Department, Peshawar: 3/5-SOPT(HD)2019—No 5230-33/55/C/Vol.60)). The duration of the experiment from beginning to end (instructions, consent, trials, voice ratings, real music ratings, harmonisation ratings, debriefing), was 1h 30mins on average.

Informed consent was a pre-requisite to begin the study. In the United Kingdom it was in the form of written consent implemented into the online study, whereas in Pakistan it was provided verbally and recorded through an Olympus VN-5500PC digital voice recorder. Throughout the procedure, local interpreters were always present to translate in the participants’ own language and in terms meaningful to them, due to the large number of non-literacy among the population in the Chitral district. This was done through personal contacts of the first author, who had established relations with a local interpreter with considerable research experience through collaborations with researchers in linguistics in the past. Verbal consent was considered the most appropriate course of action, as participants would have been alienated to the task should written consent be asked for when they are at a non-literate stage, and also not part of their cultural practice. Participants who provided consent were given a detailed description as to how the experimental procedure would unfold, were briefed at the end and compensated for their time. Participants were presented with cut-outs of the SAM model’s [54] modified images in random order, and were asked to place them in order of magnitude after the words valence, energy and dominance were explained to them through the assistance of the onsite translator and in terms meaningful to the participants’ own cultural norms. Bradley and Lang refer to pleasure, arousal and dominance in SAM, but the translation for participants in Pakistan was closer to *energy*. Considering that several music and emotion studies use arousal and energy as equivalent terms [56, 57], we prefer to use energy here. The terms were defined as valence being the pleasantness of the music stimulus (how pleasant or unpleasant it is), energy arousal as the intensity of the music stimulus (how calm or how energetic it is), and dominance as the perceived degree of control exerted by the music stimulus (how inconspicuous or dominant it is). The cutoff score at this stage was a maximum of two errors (for all dimensions) on their first trial, and no errors permitted on the second trial. The success rate at this stage was 100% for all groups. After passing the dimensional assessment, the participants were presented with an image containing 16 portraits from the MSFDE database [55] (see SI) and were asked to select which image looks like a member of their cultural group. A pilot run had previously indicated the two most likely portraits to be perceived in terms of physical appearance as members of the Kalash and Khow tribes. This primary selection was verified during experiment proper, with overwhelming preference towards one portrait by both groups (MSFDE portrait number: 252-00). This stage took place only in northwest Pakistan.

Participants were then shown four emotions from the database (anger, joy, sadness and fear) separate from each other and in random order of presentation. At this stage two cutoff scores were implemented: 1) the assignment of a positive emotion to a facial expression with negative valence, or 2) failure to make a congruent association of facial expressions of happiness, sadness or anger with any synonymous word (e.g., for sadness, terms such as ‘sad, unhappy, crying,’ would be accepted, after consulting with the onsite translator). Though no mistakes were permitted, all participants were successful from all groups. The next stage involved listening to voice recordings in Urdu (presented always first, and in random order of presentation from the Urdu voice database [58], and then in German [59]) in random order of presentation and always different from the Urdu order. Here, participants were asked to use the MSFDE and SAM modified rating scales to access the voice recordings as to their emotional content. The instructions given to the participants (in their own respective languages were to “Listen to each sample and assess which emotion(s) you think the speaker (later on: music) is expressing (if any), on the following scale: 0—no emotion at all, to 5—highly indicative of that specific emotion. Then, indicate whether the speaker (later on: music) is expressing high or low valence, energy & dominance”. If participants from northwest Pakistan failed to use the valence and energy scales in congruent manner with the voice recordings for Urdu (a language they were familiar with) they would be omitted, whereas for German speech any response was permitted. Again at this stage all participants were able to make congruent associations between Urdu speech and its intended emotional content. The majority of the participants were successful in providing congruent responses to the assessment tests (see S1 Appendix, S1 Fig in S1 Appendix for detailed results regarding the voice recordings, and and S2 Fig in S1 Appendix for the real music stimuli), even when said speech and music were unknown to the participants’ own cultural norms.

Participants then rated real music samples from their own culture which had clear emotional content. Further, music samples were included from a validated database [60], as well as samples from other cultures as well. The samples were fully randomised. Participants who failed to associate in congruent manner samples of their own music with their emotional association were not permitted to proceed to the main part of the experiment. As such, three participants were removed at this stage. These pre-assessment tests were put in place so as to ensure that participants from north west Pakistan became familiar with 1) the concept of participation in an music psychology experiment, 2) the proxy rating scales (e.g., that an image of a person smiling may stand for a piece of music which has positive valence, a mannequin figure which is seen as dancing may represent a piece of music rated high in energy), and 3) the notion of rating speech and music itself, in terms of its emotional content.

After the pre-assessment tests, the participants took part in the experiment proper, where they listened to the different harmonisations of melodies in four pseudo-randomized pre-recorded orders of presentation. At the end of the experiment, participants in all fieldwork locations were debriefed and fully compensated for their time.The average duration of each session was approximately 90 minutes long. This included explaining the scope of the study to the participants through the translator, acquiring their consent, familiarising with the equipment and the methodology, the pre-assessment tests and the experiment proper.

### 2.2 Participants

Prior the experiment, we estimated the sample size based on moderate effect size (0.30) as a reasonable compromise of the large effect sizes reported in [4, 5] with more substantial musical manipulations (dissonance manipulations or real excerpts expressing different emotions). With a significance level of 0.05 to obtain a power of 0.80 for a comparison of three groups, 37 participants are needed in each group.

#### 2.2.1 UK participants

101 participants from the United Kingdom contributed to the research, 75 of whom were female. All were native English speakers (English monolinguals—100%), with a mean age of 35.0 years (SD = 12.81). 30 were practicing musicians of Grade 5 level or equal practice-based experience, and 14 were of Grade 8 level or above.

#### 2.2.2 Kho participants

34 Kho participants contributed to the research, all of whom were male. All were native Kho speakers, with a mean age of 25.6 years (SD = 5.60). Participants were multilingual: 100% were fluent in Khowar and Urdu, and 88.2% were fluent in Pashto. Other languages spoken were: Kalash, Gujuri, Punjabi, Nuristani, English, Arabic, Hunza. Kho participants professed a music performance-based experience of M = 7.16 years. Most common performance instruments included drums (gilliken, dumduma, doll), group and solo singing, Chitrali setar, the Chitrali flute (belu). The participants expressed total unfamiliarity with any form of Western music (from the real music styles played to them in the pre-assessment stage) and any form of music notation. Due to local cultural customs, it was not possible to recruit any female participants among Kho speakers.

#### 2.2.3 Kalash participants

34 Kalash participants contributed to the research, out of whom 4 were female. All were native Kalash speakers, with a mean age of 23.7 years (SD = 4.67), and multilingual: 100% were fluent in Kalash, Khowar and Urdu, and also 47.06% were fluent in Pashto. Other languages spoken were Punjabi, Gujuri, English, Nuristani, and Hindi. The Kalash participants professed a music performance-based experience in music of M = 8.53 years. Most common performance instruments included drums (gilliken, dau, wac), singing in groups and in solo performance, and the Kalash flute (ispnoe). The participants expressed total unfamiliarity with any form of Western music (from the real music styles played to them in the pre-assessment stage) and any form of music notation.

## 3 Results and discussion

The data analysis strategy including the diagnostics of the self-report scales and validation of the concepts and tasks is provided at the S3 Appendix.

Our main aim was to explore group differences across the different harmonisation styles. To contextualise the similarities or differences between the three groups in this task, we first explored the variability within and between the groups. Such analyses tend to suggest that many musical features or behaviours vary more substantially within cultural groups than between those groups in large analyses of musical materials from all around the world [1, 53]. We utilised the coefficient of variation (CV) to index this variability for each emotion concept, shown in Table 1. This paints a similar picture to past studies where a larger variability is observed within the cultures (overall *CV*_*Within*_ = 36.4 − 40.0 vs *CV*_*Between*_ = 1.0 − 7.8). In other words, UK, Kalash, and Kho participants exhibit overall more similar responses to the whole set of stimuli and the same holds for the individual harmonisation styles in comparison to the higher variability that exists within each group. The one notable exception is valence for the whole-tone harmonisations where variability between the groups is on the same level as the variation within the groups. Having corroborated the pattern of variability is higher within the cultural groups than between those groups [1, 53], we turn our attention to the means of the self-report scales for each emotion and harmonisation style.

**Table 1:** Variability between and within cultures overall and for each harmonisation type using coefficient of variation (CV).

Excerpt	Valence		Energy		Dominance	
	Between	Within	Between	Within	Between	Within
Overall	1.0	36.4	7.8	37.8	2.7	40.0
Solo	9.2	30.8	11.7	40.7	6.8	42.4
Organum	12.5	34.4	14.3	38.3	9.4	40.2
Bach-Maj.	11.3	28.4	5.5	33.7	6.5	37.7
Bach-Min.	5.7	31.5	6.8	35.4	13.4	37.3
Jazz-Maj.	5.4	32.1	5.8	35.0	7.1	36.8
Jazz-Min.	2.9	33.5	7.6	35.3	12.4	35.9
Epirote	23.1	33.9	17.6	36.1	18.6	34.9
Kalash	7.4	39.7	22.7	40.9	26.2	36.6
Whole-tone	41.2	38.7	9.1	30.9	16.5	30.1

**Fig 1:**
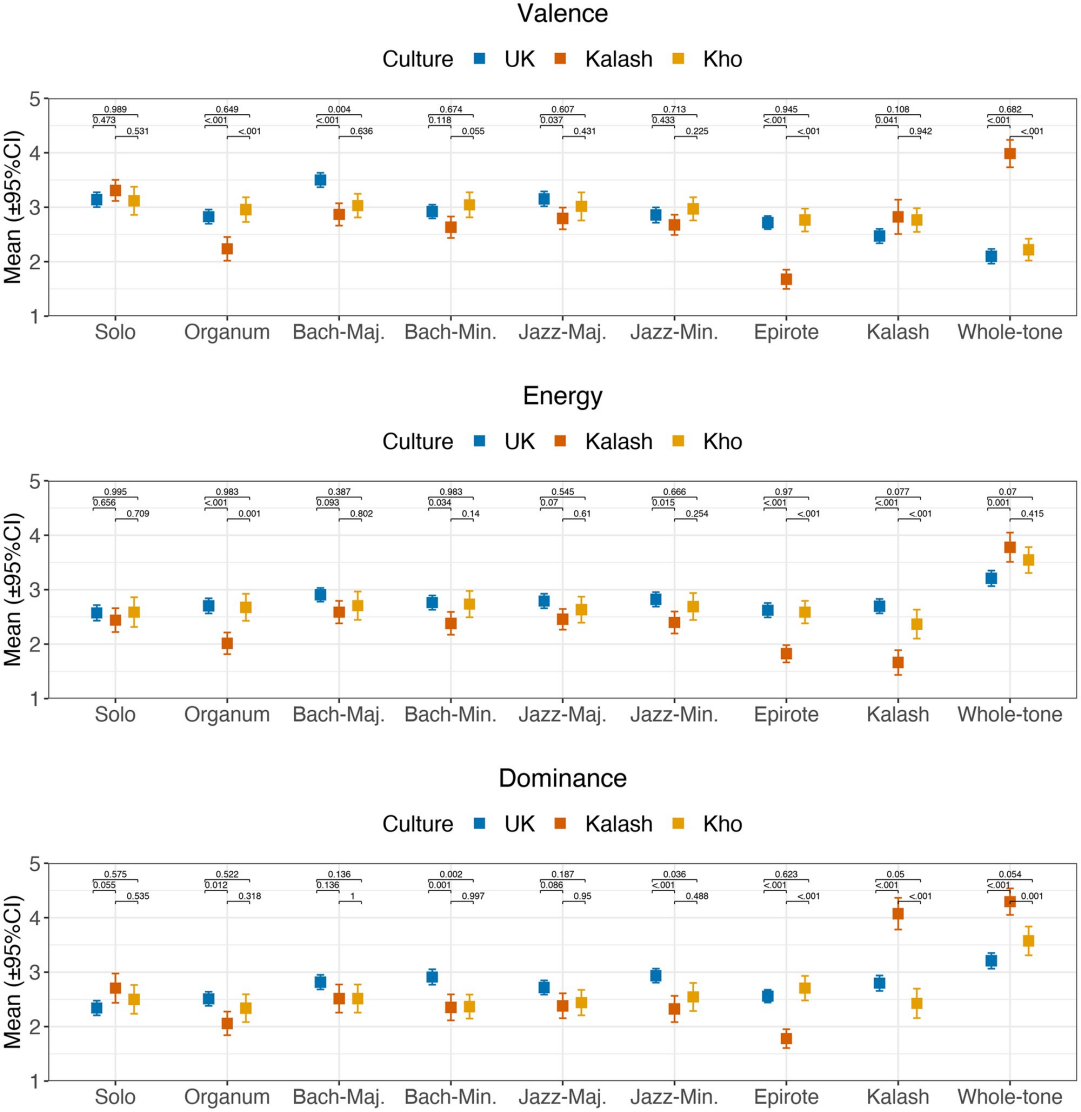
Ratings of three affect dimensions across harmonisation styles for each cultural group. The p-values indicate the results post-hoc contrasts between the three groups within each harmonisation style adjusted for multiple testing (Tukey’s method). Error bars denote 95% confidence intervals.

For the subsequent analyses of the possible differences in the self-report ratings of emotions, we utilised generalized linear mixed models with four random factors (participant, melody, note density, and melodic range) and two fixed factors (Culture with three levels and Harmonisation style with nine levels). The two musical variables, note density (number of onsets/sec) and melodic range (difference between the highest and lowest pitch in semitones) were included to control the differences created by the harmonisations (see S1 Appendix for descriptions). The mean ratings of the emotion dimensions across all participants is shown in Fig 1.

In broad terms, ratings across the three dimensions (valence, energy, and dominance) showed significant differences across Culture and Harmonisation style; For valence, significant main effect of Culture (*t*(3042,169) = -2.82, *p* = .005) and Harmonisation styles (*t*(3042,169) = -6.96, *p*<.001) was observed and these two factors also exhibited a significant interaction (*t*(3042,169) = 4.26, *p*<.001). For the ratings of energy, the overall pattern is somewhat different; no significant main effect of Culture (*t* = -1.66, *p* = .098) but significant effect of Harmonisation style (*t* = -2.76, *p* = .0088), and no significant interaction between the two (*t* = 0.79, *p* = .428). For dominance, significant main effects were observed for Culture (*t* = -2.88, *p* = .0042) but not for Harmonisation style (*t* = -0.64, *p* = .525) but these two factor interact (*t* = 3.05, *p* = 0.0023).

In our first hypothesis, we predicted that Western harmonisation styles would fail to elicit similar responses from non-Western participants, when compared to the responses from the Western participants. UK participants found Bach major and Jazz major harmonisation styles generally more positive than Kalash and Kho participants (valence for Bach, M = 3.50 compared to Kalash M = 2.87, *t* = -4.34, *p*<.001, and Kho M = 3.03, *t* = -3.23, *p* = .0037 and for Jazz, M = 3.15 compared to Kalash M = 2.68, *t* = 2.47, *p* = .0368, and Kho M = 3.01, *t* = 0.95, *p* = .607), which is for the most part in line with the hypothesis. We also predicted that we would observe differences between the groups with respect to mode. If we look at the ratings of valence for all groups with respect to two harmonisation styles that had major and minor versions (Bach and Jazz), the ratings of UK participants to excerpts in major mode were significantly higher (M = 3.33) than for the excerpts in minor (M = 2.89, *t*(1180) = 3.88, *p* = .0074). In contrast, the ratings of the Kho participants were not influenced by Mode (minor M = 3.01, major = 3.02, *t* = 0.106, *p* = 0.917) nor Kalash participants (minor M = 2.83, major M = 2.65, *t* = 1.27, *p* = 0.220). A similar pattern of results is mirrored by the ratings of dominance, and also for basic emotions (anger, fear and happiness, see S5 Appendix). In sum, the distinction of mode communicates emotions to Western participants but not to non-Western participants.

The second hypothesis, which is a non-Western variant of the first hypothesis, postulated that familiar harmonic idiom will be sufficient to create differences in emotional expression in non-Western participants. Here we had one harmonisation style that was appropriate for one of the non-Western participant groups, the Kalash drasailak harmonisation style. Indeed, Kalash participants consider their own harmonisation style to be significantly lower (M = 1.66) in terms of energy compared to Kho (2.37) and UK (2.70) participants (*t* = -6.81, *p*<.001 for the contrast between Kalash and UK, and *t* = -3.79, *p*<.001 between Kho and UK). Also, Kalash participants allocate high dominance (M = 4.07) to their own harmonisation style. In comparison, Kho (M = 2.43) and UK participants (M = 2.80) provide significantly lower ratings for these examples (*t* = 8.52, *p*<.001 for the contrast between Kalash and Kho, and *t* = 8.08, *p*<.001 between the ratings of Kalash and UK participants). This is a reassuring observation and suggests that the Kalash found this harmonisation style familiar and recognised it as something that they could relate to, and the ratings are in line with the second hypothesis.

As a variant of the second hypothesis, we looked at the differences related to the whole-tone harmonisation style. In theory, this should be an unfamiliar harmonic idiom to all groups. This was generally perceived as unpleasant by the UK participants (valence M = 2.13) and Kho (M = 2.25) participants but—surprisingly—pleasant by Kalash participants (valence M = 4.01, *t*>9, *p*<.001 for both contrasts). Also, the Kalash participants consider the whole-tone harmonisation style to have particularly high levels of energy (M = 3.78), and although this is not statistically different from the Kho participant ratings (M = 3.54, *t* = 1.27, *p* = .416), the ratings of Kalash participants differ significantly from the ratings given by the UK participants (M = 3.21, *t* = 3.76, *p* = .0005). For dominance, the difference between the Kalash and the others is also noteworthy; The whole-tone harmonisation style received significantly higher ratings from the Kalash participants (M = 4.31) in comparison to those given by Kho (M = 3.59, *t* = 3.73, *p* = .0006) and UK participants (M = 3.22, *t* = -6.88, *p*<.001). These observations would be in line with the second hypothesis if we assume that the Kalash are regarding the whole-tone style as a familiar idiom, which we did not assume, but a more nuanced interpretation of this will be attempted in the Discussion.

**Fig 2:**
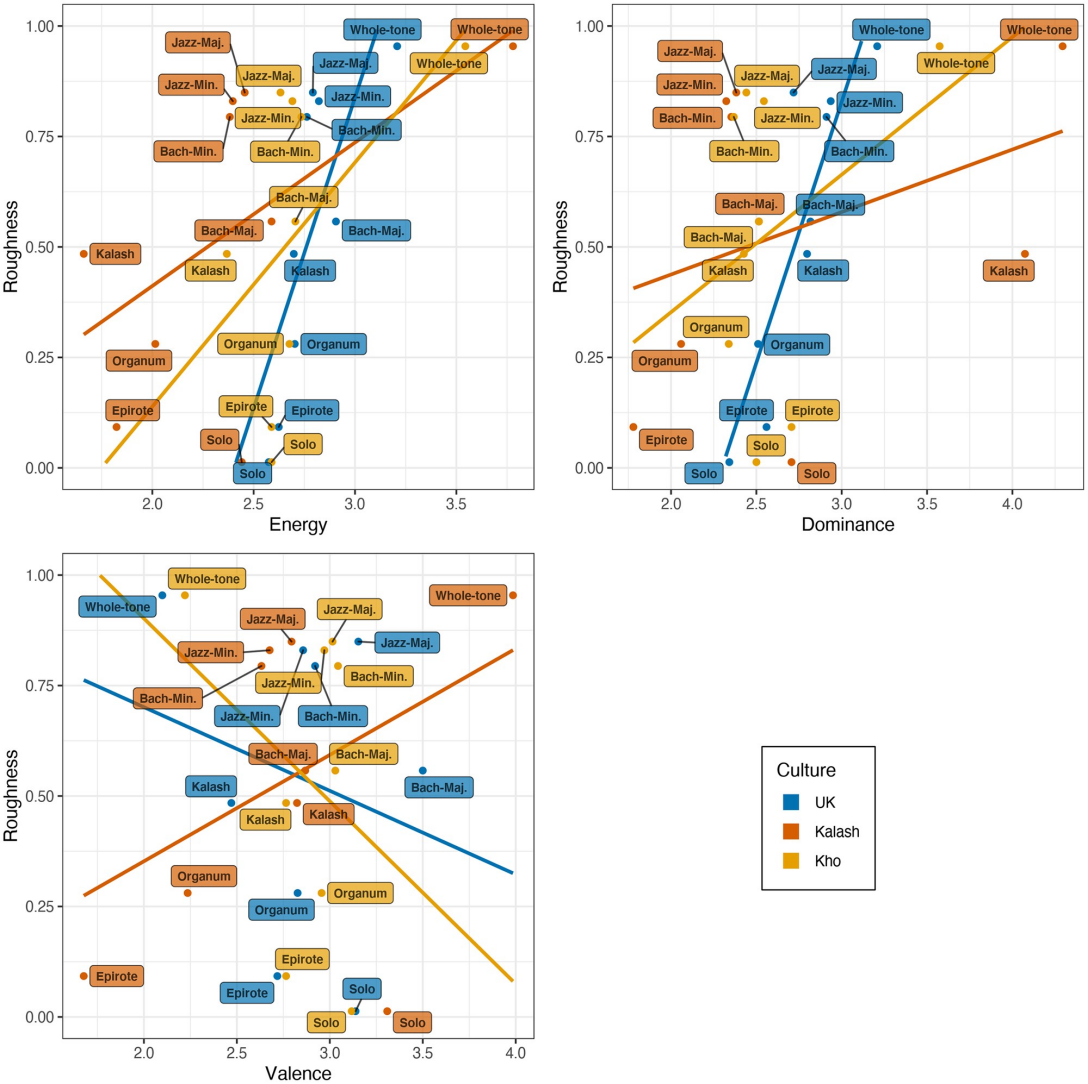
Ratings of the three emotion dimensions and acoustic roughness across the three groups.

Finally, our third hypothesis postulated that acoustic roughness would play a distinct role in emotional expression. Based on past literature linking harmony perception with acoustic correlates, we predicted that higher roughness would generally lead to higher ratings of energy [51] and consequently dominance, and lower ratings of valence [48, 61]. Such a broad associations were observed in the data collapsed across the stimuli (Fig 2). To explore the association between roughness with emotion ratings more thoroughly, we correlated the individual emotion ratings for all stimuli and the output of a sensory roughness model [62] for all stimuli using a rank correlation measure (Kendall’s *τ*) due to non-normally distributed rating data. For dominance, a linear relationship between roughness and dominance emerged (*τ* = 0.180, 0.183, and 0.114 for UK, Kalash, and Kho participants, all *p*<.005). A similar positive association holds for roughness and energy (*τ*(907) = .144 *p*<.001 for UK, *τ* = .290, p<.001) for Kalash, and (*τ* = .148, *p* = .001) for Kho participants. Although the coefficients were rather small, the results of this correlation analysis from these emotion dimensions was in line with our hypothesis 3, which was founded on the assumption that higher roughness is typically related to more perceived energy [51] and evidently also to more perceived dominance. Finally, valence and roughness exhibited a negative relationship, *τ* = -0.08, *p*<.001 for UK and *τ* = -0.105, *p* = .016 for Kho participants, but inverse pattern *τ* = 0.220, *p*<.001 for Kalash participants. The reverse link between roughness and valence also supports our hypothesis but the positive association between valence and roughness for Kalash participants was against the pattern and most likely linked to a different aesthetic values that also led them to positive valence ratings for the whole-tone stimuli.

In our first hypothesis for this study, the prediction was made that Western concepts of harmony will not be relevant for participants unexposed to Western music when other emotional cues are kept constant, including the major/minor distinction between samples. This was largely supported by the data. Regarding the second related hypothesis, it was demonstrated that harmonic style alone has the ability to colour the emotional expression in music if it taps the appropriate cultural connotations. This was supported in terms of the Kalash participants’ ratings for the Kalash harmonisation style, as well as for the British participants’ ratings for the major and minor modes of the Bach chorale and Jazz harmonisations. Finally, we hypothesised that acoustic roughness may influence the perception of emotions in the stimuli. This hypothesis received support in the form of significant correlations between roughness and the dimensional ratings in particular.

To summarise, the data presented here attest both cultural specificity and universality in communicating emotions through music. First, preference for one musical (harmonisation) style over another is dictated by culture as a means of exposure—the Kalash tolerance for dissonance is evident of this trend, but it is necessary to clarify that the tribe’s music is very diverse, and despite the fact that some of their ritual music contains a high level of roughness, their non-ritual music is consonant. Therefore, preference for consonance seems to be dependent on familiarity, which is evident by i) a clear preference of the Kalash tribe for the whole-tone harmonisation style (as provided by the automated transcription software—see Stimuli section in the S1 Appendix) in the conditions of this experiment, and ii) to their own style of harmonisation (which they allocated as having low energy and of moderate valence, but rated it very high in terms of dominance). Their responses can be justified if we take into account that they have recognized the harmonisation style as Kalash music, and as such, rated it accordingly based on the familiarity effect. This particular harmonisation style used is the drasailak dance (homophonic drones sung as chromatic clusters), characterized by its slow tempo and somewhat sombre delivery. A similar observation has been made by Messner [50] who found highly dissonant two-part singing to be aesthetically appreciated in Papua New Guinea.

Regarding the two melodies utilized in the study these were not exceptional in any way in terms of emotion elicitation; this was established during a pilot trial with Western participants, and members of the tribes living in Europe. Though both melodies appear to be distinctly Western, this was not the case. The second melody was developed having in mind Kalash vocal performance practice used in the Chawmos Winter festival, in which a large corpus of the melodies sung are in the pentatonic scale. Regarding the Khow tribe, the melodies developed would not typically be identified directly as representative of their own musical style—however, they are not completely alien to them. Kho tribesmen are partly exposed to Urdu pop, which, in principle, follows western diatonic harmonisation principles. This means that knowledge of this melodic style is implicit, and not direct. Nevertheless, it has to be acknowledged that the melodies themselves posed a certain restriction, and there is certainly a need for further research utilizing authentic melodies from the tribes’ own repertories so as to avoid having any doubts regarding the veracity of responses in the processing of the emotional content of the examples.

Correlation between roughness and perceived anger across all groups is in line with previous research linking roughness to anger in speech perception [46]. This might imply a possible universal in music perception, even if it is early to make any conclusions about this finding. What is of further interest is that previous research has also pinpointed a sensitivity to roughness perception cross-culturally, although this was not linked to the perception of unpleasantness in single chords in that particular setting [21].

The ratings of valence, energy and dominance, in addition to the ratings of basic emotions, suggest that the non-Western participants did not rate major and minor modes significantly different from each other in this experimental setting. This is in contrast to the British participants who rated major and minor harmonisations differently from each other, as was expected (see 1st hypothesis results).

Even though the expressions of a wide range of emotions (basic and complex) is inseparable from both Khow and Kalash performance culture, and based on the data presented here, mode (major, minor or the local Chitrali modes) does not appear to stand out as the most significant part in this equation (for more information on local performance culture, see SI). Although this distinction has a very important role in Western music, our data suggests that there is no clear evidence for the major/minor effective convention with either of the Pakistani tribes, as the emotional connotation of the songs, is rather determined by core musical parameters (style, tempo, loudness, pitch height), as well as a variety of extra-musical parameters (performance setting, lyrics, instruments, identity, age and gender of the performers). This finding may place doubt on the notion that specific modes are de facto associated with specific emotions in a universal manner, without carefully considering all the parameters involved [5]. This viewpoint also falls in line with the finding that in Western musical culture tempo is mastered earlier than mode to judge the emotional tone conveyed by music [31], suggesting that the major-happy minor-sad distinction is a learned cultural convention instead of an innate musical universal.

## 4 Conclusions

Our research points towards that, to some extent, there may be a universal propensity to agree that some emotional evaluations towards specific sonic events may be considered as common across cultural boundaries. We have presented newly found evidence that a harmonisation style with a high level of roughness, yet with similar levels of loudness, timbre, tempo and harmonic rhythm as the rest of the stimuli, is considered to convey energy, dominance and in particular, anger—even when participants have had little prior exposure to similar stimuli, and were divergent in terms of background culture. At the same time, our analysis concurs with past research that the perceptual variability of behaviours linked to musical practices within groups is more substantial than the variability observed between groups—in this case, regarding the emotional content of harmonisations of simple melodies. It should be noted, however, that our study is by no means intended as an overview of all possible diverse or similar responses to the issues that we have raised; we acknowledge that the manner in which harmonic variations are experienced, primarily in relation to dissonance, may vary widely within and between cultures originating from other parts of the world and dependent on a number of issues. The current paper provides a reference point which addresses the assessment of how, while maintaining the same melody, overall harmonisation accompaniment possesses the ability to convey distinct emotional meaning to listeners, while at the same time acknowledging that this is a complex process involving both aspects of familiarity brought about by enculturation, as well as common cognitive processing operating in similar manner across cultures.

## Supporting information

S1 Appendix Stimuli. Description of the speech and music examples for the validation of the rating scales as well as the harmonisation stimuli.
(PDF)

S2 Appendix Self reports. A summary of the two different self-reports methods, one relying on a self-assessment mannequin [54] and another based on facial expressions of emotion [63], that were adapted to the study.
(PDF)

S3 Appendix Data analysis operations. A summary of the data analysis operations carried out before the main analysis operations including the consistency analysis of all ratings.
(PDF)

S4 Appendix Ethics, practical considerations and additional information. A summary of ethical considerations before entering the field, including a concise briefing as to the status, role and presence of music among the two tribes in northwest Pakistan.
(PDF)

S5 Appendix Additional analysis operations. A report on how the harmonisation stimuli were analysed in terms of basic emotions.
(PDF)
