# Correction: Harmonic organisation conveys both universal and culture-specific cues for emotional expression in music

**DOI:** 10.1371/journal.pone.0264048

**Published:** 2022-02-10

**Authors:** George Athanasopoulos, Tuomas Eerola, Imre Lahdelma, Maximos Kaliakatsos-Papakostas, Ljubica Damjanovic

Ljubica Damjanovic should be included in the author byline. Ljubica Damjanovic should be listed as the fifth author, and their affiliation is 3: Liverpool John Moores University. The contributions of this author are as follows: Conceived and designed the experiments, drafted the article for important intellectual content, provided final approval of the version to be published.

The correct citation is: Athanasopoulos G, Eerola T, Lahdelma I, Kaliakatsos-Papakostas M, Damjanovic L (2021) Harmonic organisation conveys both universal and culture-specific cues for emotional expression in music. PLoS ONE 16(1): e0244964. https://doi.org/10.1371/journal.pone.0244964
