# Supplementary material for: Harmonic organisation conveys both universal and culture-specific cues for emotional expression in music
Source: PLoS One. 2021 Jan 13;16(1):e0244964. doi: 10.1371/journal.pone.0244964 (PMC7806179; doi:10.1371/journal.pone.0244964)
Supplement: S1 Appendix — Description of the speech and music examples for the validation of the rating scales as well as the harmonisation stimuli. (PDF) [file pone.0244964.s001.pdf]

S1 Appendix

Stimuli

To ensure that participants are able to use the self-report instruments, we first utilised well-known stimuli from expressive speech research and real music stimuli before turning out attention to harmony. The purpose of the speech and real music were to familiarise the participants with the task and self-report instruments.

Speech stimuli Six Urdu [1] and six German speech excerpts [2] representing anger, joy, and sadness were used (two different excerpts for each emotion). The ratings may be seen in S1 Fig: Means of self-report scales for speech.

S1 Fig. Means of self-report scales for speech.

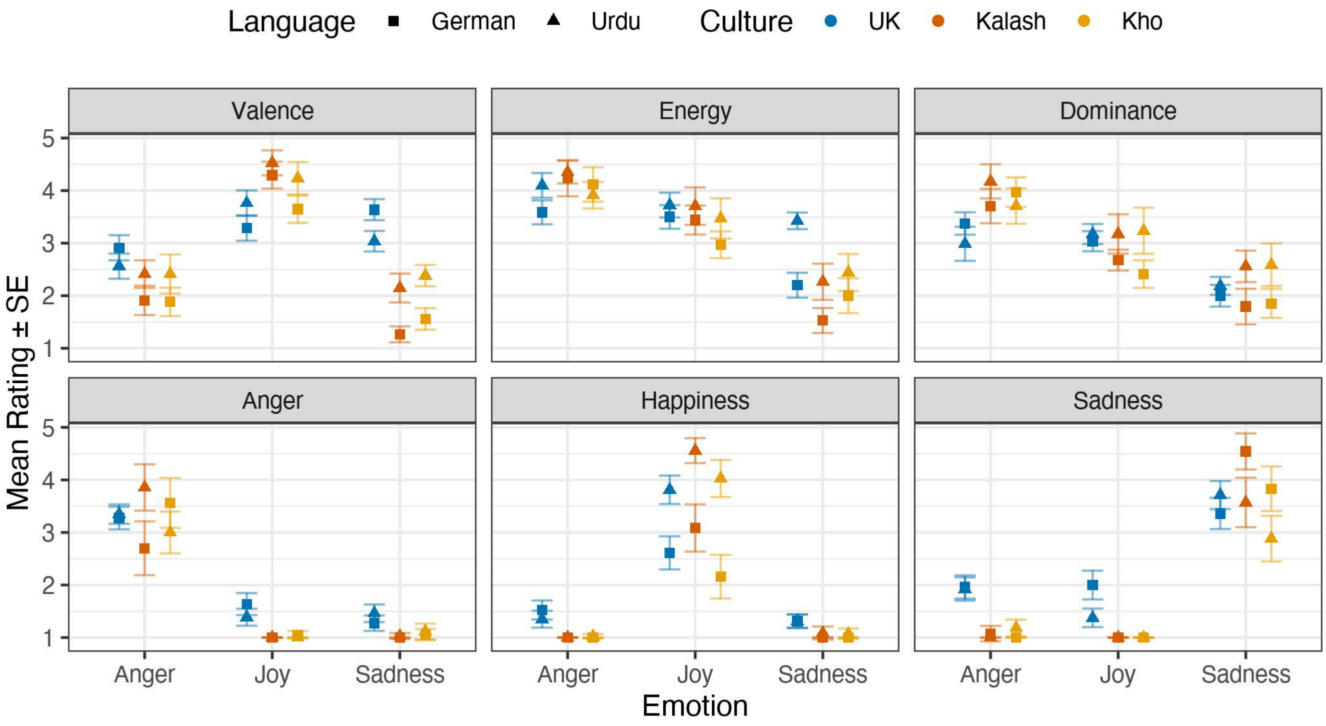

Real music Ten music examples in total were used, which included excerpts taken from Western music (4; these included Western art music from the classical era, contemporary film soundtracks, 1950s west coast Jazz, Death metal), rural Pakistani music from Chitral (3; these included Kalash major pentatonic music from the Chawmos festival, Kalash dražailak drone chant music from the Zoshi festival, and Khowar mahfil music), and lastly Moroccan (Berber) music, Indian disco music, and Greek Epirote polyphonic music. The real music samples were used to probe the same emotions represented by the speech examples (joy, sadness, and anger) as well as examine how participants would evaluate the samples in terms of dimensional ratings (see Table 1).

Table 1. Pre-assessment stimuli, target emotions and roughness calculations.

Name	Details	Emotions	Roughness
Jazz	Chet Baker, Almost Blue, 1987	Sadness	0.000
Film music	Example 031 from film soundtrack dataset [3]	Sadness	0.013
Kalash chromatic drone chant	Kalash vocal music (ghak) from Zoshi festival	Sadness	0.451
Epirote lament	Epirote polyphonic singing in Paramythia, Epirus, Greece	Sadness	0.483
Western classical	G. Rossini, excerpt from the Barber of Seville	Joy	0.704
Moroccan dance	Moroccan street musicians in Essaouira, Morocco	Joy	0.818
Indian disco	Saranjit Singh, 1982	Joy	0.853
Khowar	Khowari mahfil performance by Mansoor Ali	Joy	0.849
Kalash major pentatonic	Kalash vocal music from the Chawmos festival	Joy	0.937
Death metal	Death by Misanthrope	Anger	1.000

The excerpts selected by the authors were done so as to include a variety of musical genres of Western and non-Western music in terms of style, which portrayed significant variance as to their emotional content both in terms of the most common emotions found cross-culturally, as well as in terms of emotional dimensions in Russell's model [4]. The real music stimuli used were relatively unknown to the average music listener, but nevertheless indicative of their particular musical genre. The purpose of this multi-varied approach was to ensure that the real music samples used would not run the risk of being recognized, and to the best of our abilities, omit any personal associations that the participants may have with the stimuli through episodic memory.

Kalash and Kho participants were asked if they were familiar with the real music stimuli. While they recognised the origin and performance setting of the Kalash and Khow music stimuli, they were completely naive to all Western stimuli. Although participants were asked whether they "liked" the music stimuli, these results cannot be taken into consideration as i) valence ratings were already being obtained ii) a question of this type may provide false data due to the novelty effect [all new music would be viewed favourably], and even possibly iii) participants could have provided false positive responses if they believed that this would be viewed favourably by the researcher. Ratings of the real music stimuli may be seen in S2 Fig: Means of self-report scales for real music stimuli.

S2 Fig. Means of self-report scales for real music stimuli.

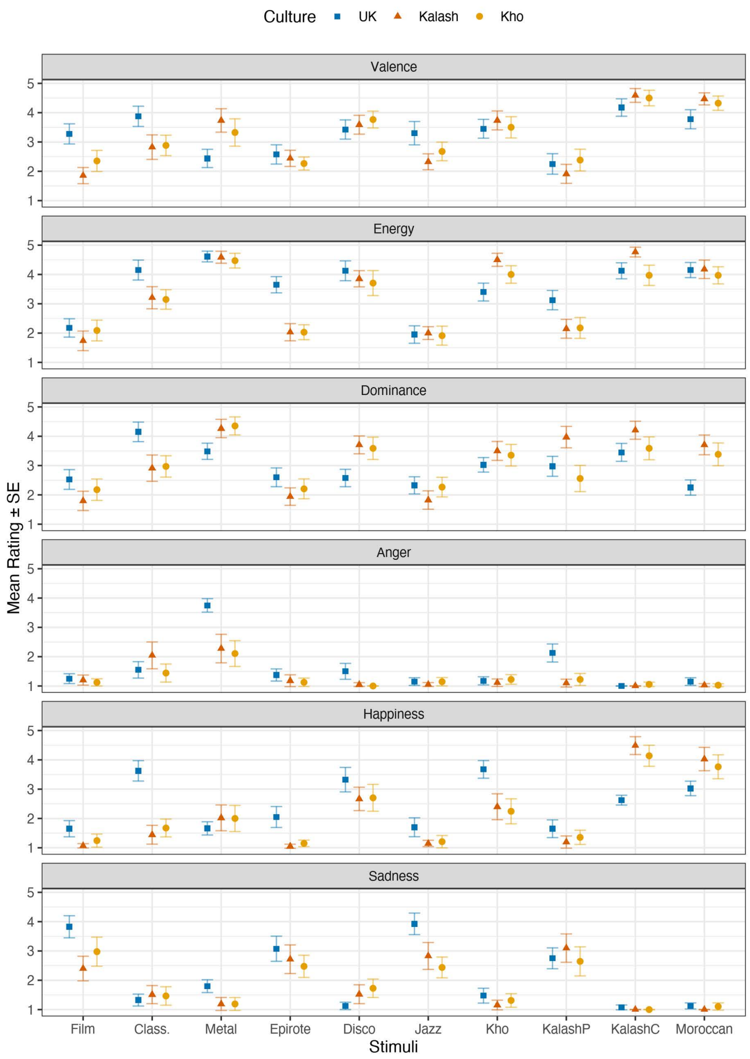

Dimensional ratings by the participant groups of the real music stimuli and their roughness levels may be seen in S3 Fig.

S3 Fig. Scatterplots of self-report scales for real music stimuli and roughness.

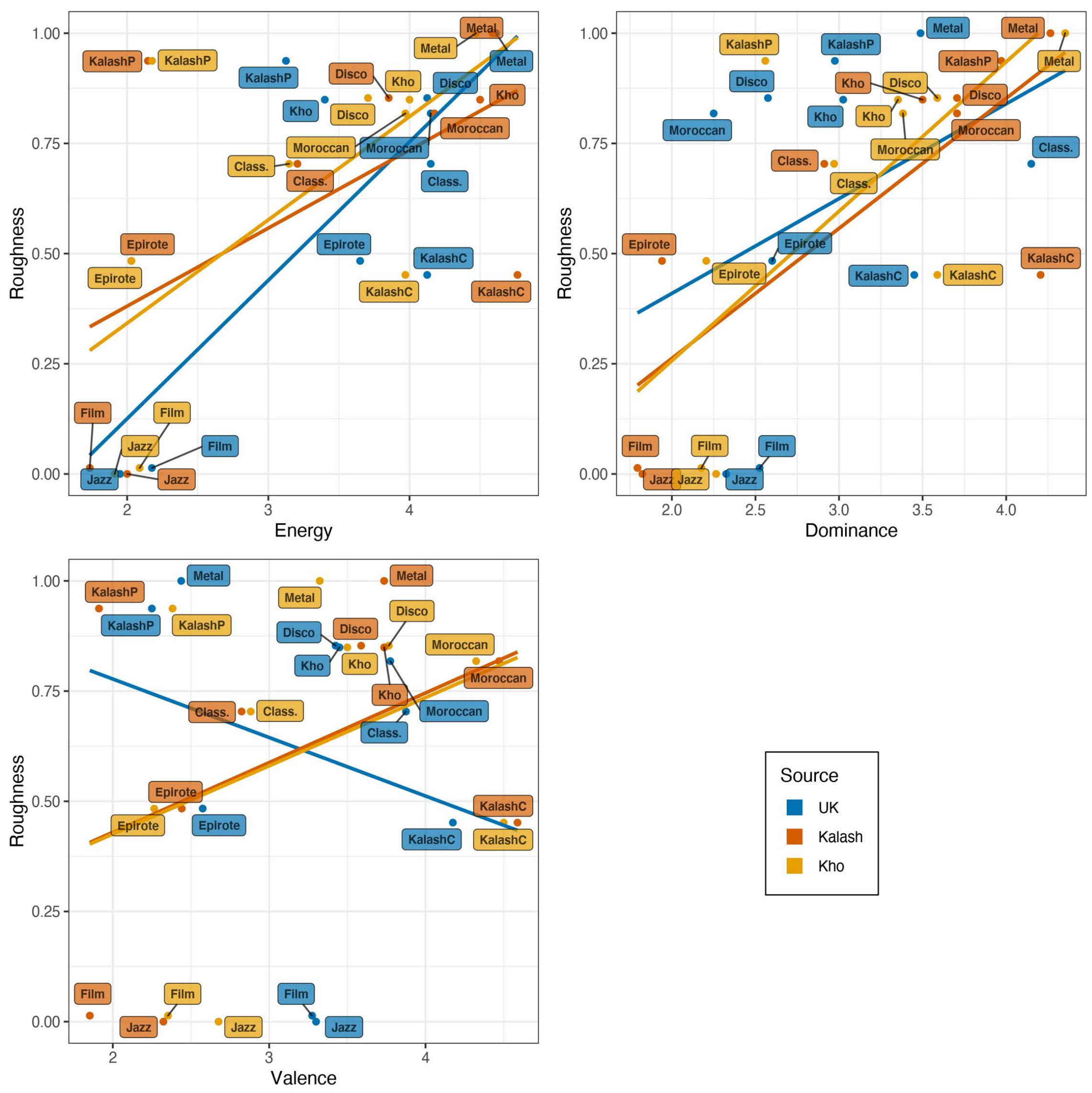

Harmonised Melodies Two melodies were chosen as the core skeleton for the harmonisation. The selection of melodies were notated using Sibelius 8 music notation software [5]. Each melody was approx. 15 seconds long. The melodies chosen were not exceptional in any way in terms of eliciting a specific emotion. This was addressed during a pilot trial with Western participants of mixed origin in Durham University, and with Kalash participants living in Europe. They were harmonised with the *Chameleon melodic harmoniser* software (see http://ccm.web.auth.gr/chameleonmain.html), which can (re)harmonise any melody into different genres of musical style providing the experimental stimuli for the proposed work. The two melodies, which did not have an implied modal (major/minor) or harmonic content, were harmonised in eight different genres/styles for a total of 18 samples; 2 solo melodies and 16 variations. The harmonic variations developed were: Organum (early Middle Ages), Bach chorale maj/min(first half of the 18th century), Jazz maj/min(1914-1929), whole-tone scale (post 1910s), Epirote (Greek non-Western), and Kalash dražailak (Pakistani Kalash non-Western).

S4 Fig. Melody 1 Melody 1 and its harmonisations into different styles.

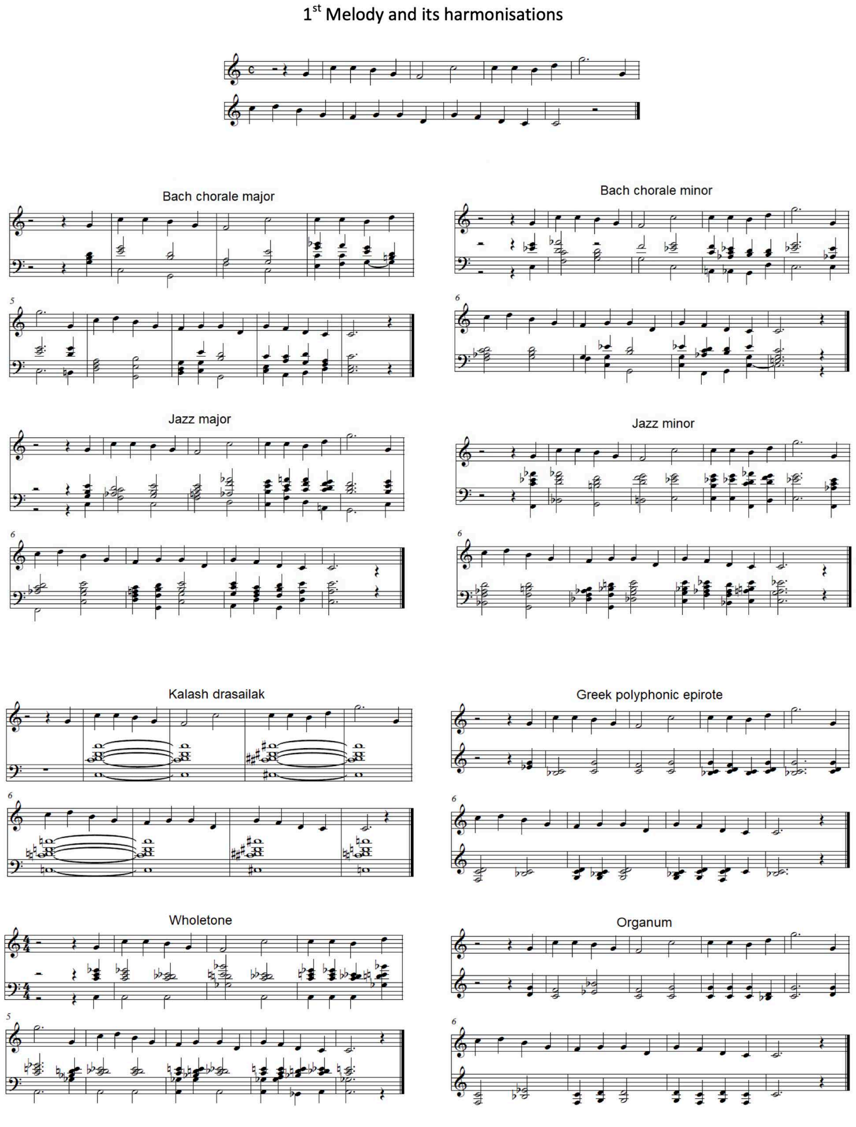

S5 Fig. Melody 2 Melody 2 and its harmonisations into different styles.

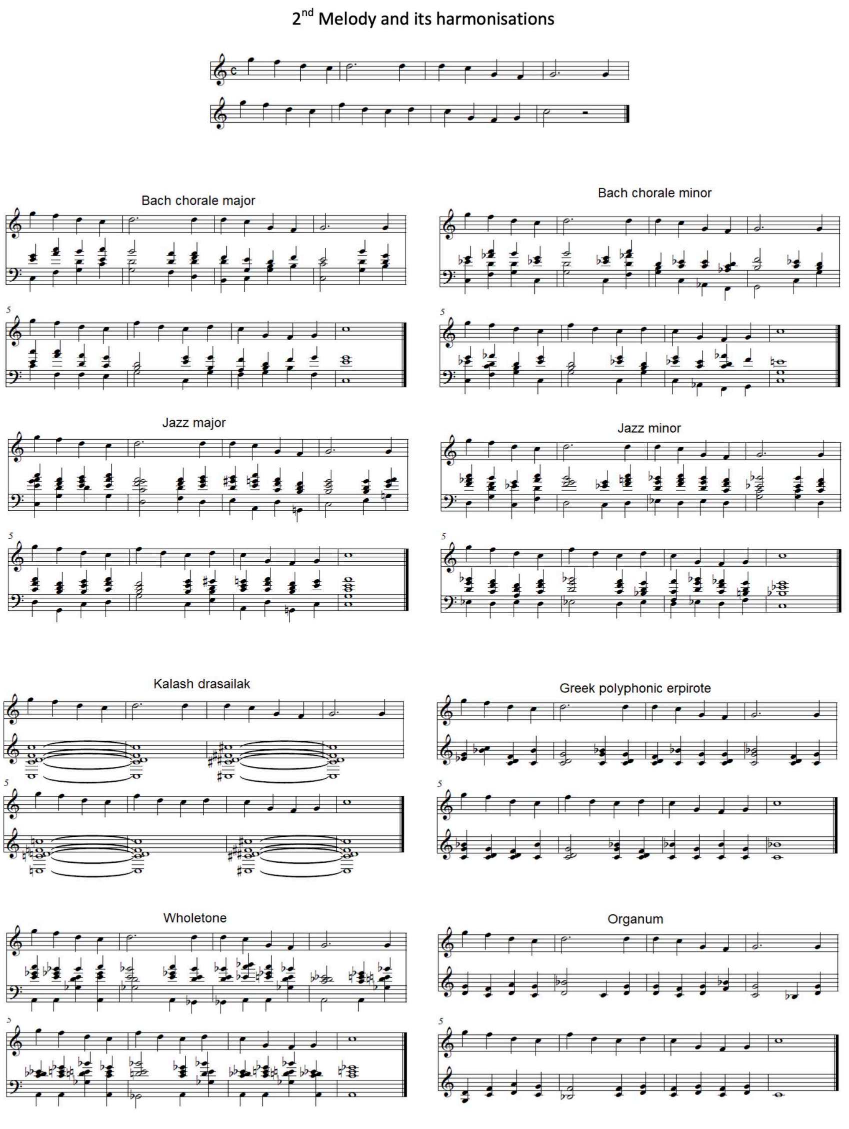

All harmonic styles were developed based on the corpus library of Chameleon itself, with the exception of the Kalash dražailak style which was developed based on the instructions of Kalash informants living in Europe, and then piloted to check their validity. The incorporation of these harmonisation styles were motivated by the necessity to investigate whether participants would be able to differentiate between the melodic music stimuli presented with harmonic backgrounds differing in mode (major/minor) as well as style (Western, non-Western, non-tonal, etc.). Though the harmonisations provided by the software may not always be 100% accurate, we decided against modifying them, so as to retain a level of objectivity. The final harmonisation

results were matched for tempo, timbre and dynamics, with harmony being the only variable parameter in the harmonisation of each melody. The melody volume level was +5 dB higher than the accompanying harmony.

The implementation of the harmonisation was carried out by one of the authors in collaboration with the lead author. The CHAMELEON [6] software was employed for generating harmonisations of given melodies in selected harmonic styles (described in detail below). CHAMELEON receives as input a melody to be harmonised, annotations about the harmonic rhythm (i.e. where chords should be placed) and a selected harmonic style [7] or two styles to be blended (the blending feature is not used in the paper at hand) and generates harmonisations for the given melody in the selected style with "vertical" chords with rudimentary voice leading at the positions indicated in the input file. The user can optionally indicate intermediate phrase endings (where intermediate cadences are placed) and important harmonic notes in the melody. The system learns from data diverse aspects of harmony, i.e. chords in the General Chord Type [8] form; first-order chord transition probabilities; cadences (chord pairs that end phrases); and bass voice leading through a probabilistic scheme [9] that combines information about chords, bass voice motion and melodic notes. Chord notes between the melody and the bass voice are defined according to a rudimentary algorithm that attempts to achieve balanced voicing (i.e. the smallest possible distance differences between successive vertical pitches in a chord) for each chord (see Table 2)

A piano timbre was used to render the examples from *Logic Pro X*. Full examples of the scores and audio are available from Open Science Framework, https://osf.io/wq4tp/. Extraction of sensory roughness was carried out by a model proposed by Wang [10] that relies on audio and simulates the auditory periphery and mechanics in the cochlea to assess the sensory roughness. The output values were normalised for this dataset between 0 and 1. The range spanned the highest and lowest notes in the stimuli also varies across the pieces (solo spans 16.5 semitones whereas Bach chorale version has twice the range – 38). The mean range of the two melodies is given in Table 2. Note density is another musical descriptor that varies significantly across the harmonisation styles, solo having only 1.43 notes / second but Jazz standard in Major has 7 notes / second. These values are also indicated in the Table 2 (Dens. column).

Table 2. Harmonisation stimuli characteristics.

Name	Description	Rough.	Range	Dens.
Solo	No harmonic accompaniment. No 3rds and 6ths in melody 1 and and no 3rds, 6ths and 7ths in melody 2.	0.000	16.5	1.43
Greek	Polyphonic Epirote style. No distinction between major and minor mode is made.	0.085	22.5	4.92
Organum	Organum, no distinction between mode is made.	0.284	25	4.07
Kalash dražailak	Kalash dražailak harmonisation, which uses a chromatic cluster of drone notes moving upwards by a minor second before returning to the original pitch class set.	0.501	31	2.36
Bach Chorale Maj.	Harmonisation style in major, contains very little chromaticism and ends using a perfect cadence.	0.579	34.5	5.13
Bach Chorale Min.	The harmonisation style uses the harmonic minor (raised 7th degree). Pieces end on the Picardian 3rd (major triad) cadence.	0.830	38	5.71
Jazz Standard Maj.	Harmonisation in major, contains occasional chromaticism.	0.889	37	7.00
Jazz Standard Min.	The harmonisation combines the harmonic and the natural minor scales and contains chromatic colouring.	0.868	34.5	6.80
Whole-tone	In the whole-tone harmonic style, contains a high level of dissonance.	1.000	37	6.94

References

1. Latif S, Qayyum A, Usman M, Qadir J. Cross Lingual Speech Emotion Recognition: Urdu vs. Western Languages. In: 2018 International Conference on Frontiers of Information Technology (FIT). IEEE; 2018. p. 88–93.

2. Burkhardt F, Paeschke A, Rolfes M, Sendlmeier WF, Weiss B. A database of German emotional speech. In: Ninth European Conference on Speech Communication and Technology; 2005. p. 1517–1520.

3. Eerola T, Vuoskoski JK. A comparison of the discrete and dimensional models of emotion in music. Psychology of Music. 2011;39(1):18–49.

4. Russell JA. A circumplex model of affect. Journal of Personality and Social Psychology. 1980;39(6):1161–1178.

5. Avid. Sibelius 8 music notation software; 2017. Available from: `https://www.avid.com/sibelius`.

6. Kaliakatsos-Papakostas M, Queiroz M, Tsougras C, Cambouropoulos E. Conceptual blending of harmonic spaces for creative melodic harmonisation. Journal of New Music Research. 2017;46(4):305–328.

7. Kaliakatsos-Papakostas MA, Confalonieri R, Corneli J, Zacharakis AI, Cambouropoulos E. An Argument-based Creative Assistant for Harmonic Blending. In: Proceedings of the Seventh International Conference on Computational Creativity; 2016. p. 354–362.

8. Cambouropoulos E, Kaliakatsos-Papakostas MA, Tsougras C. An idiom-independent representation of chords for computational music analysis and generation. In: ICMC; 2014. p. 1002–1009.

9. Makris D, Kaliakatsos-Papakostas M, Cambouropoulos E. A probabilistic approach to determining bass voice leading in melodic harmonisation. In: International Conference on Mathematics and Computation in Music; 2015. p. 128–134.

10. Wang Y, Shen G, Guo H, Tang X, Hamade T. Roughness modelling based on human auditory perception for sound quality evaluation of vehicle interior noise. Journal of Sound and Vibration. 2013;332(16):3893–3904.
