# Supplementary material for: Harmonic organisation conveys both universal and culture-specific cues for emotional expression in music
Source: PLoS One. 2021 Jan 13;16(1):e0244964. doi: 10.1371/journal.pone.0244964 (PMC7806179; doi:10.1371/journal.pone.0244964)
Supplement: S2 Appendix — A summary of the two different self-reports methods, one relying on a self-assessment mannequin [54] and another based on facial expressions of emotion [63], that were adapted to the study. (PDF) [file pone.0244964.s002.pdf]

S2 Appendix

Self-reports

Two self-report measures were adapted to suit the data collection in Pakistan and enable meaningful comparisons:

1. a dimensional rating of valence, arousal and dominance using a five point Likert rating scale

2. an evaluation of a few of the most common emotions found cross-culturally (joy, anger, sadness and fear) using facial expressions.

The dimensional ratings are based on the theory of core affect [1, 2], according to which musical expressivity and affective responses appear to be grounded and varying along two dimensions (valence and arousal, which range from negative to positive). A further dimension assessed was dominance, due to the fact that it offers a broad variability in terms of its semantic labeling, while at the same time it is the least variant parameter (in relation to valence and arousal) in terms of affective judgement. Before dominance ratings were to be collected, it was considered essential to clarify to the participants which member of the interaction is being rated: not the participant's emotions towards the sonic stimuli presented to them (felt emotion), but rather what the stimuli themselves were expressing (perceived emotions). Therefore, during the instructions to the participants, the authors made a point to clarify that dominance ratings were targeted towards expressed emotions by the sonic events. Still, within this definition, the broad variability in terms of dominance's semantic labeling makes it prone to be influenced by the cultural background of the participants – as the perception of what could potentially be perceived as dominant in music, would highlight any cultural variabilities or universal traits between them. We used a variation of the Bradley and Lang [3] visual rating scales for the assessment of these three dimensions (see Fig 1), a rating method which has yielded positive results for use in cross-cultural settings [4]. The images in relation to the energy assessment were modified so as to be culturally appropriate for the participants in northwest Pakistan as to how arousal is represented. These variations have been validated by both Western (E.U. mixed) as well as Pakistani participants during the pilot phase.

S6 Fig. Graphical illustration showing the three dimensional rating scales (A=Valence, B=Energy, C=Dominance).

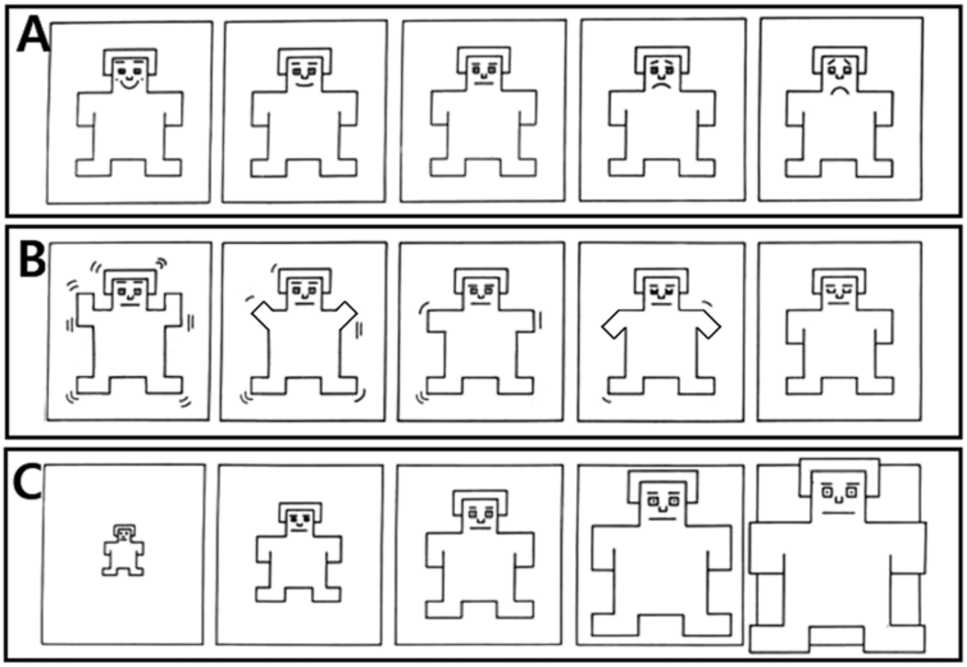

The second rating method was focused on assessing the sound stimuli in terms of potential basic emotions they expressed: joy, anger, sadness and fear are explicitly part of many non-Western theories of musical emotions [5], and Western listeners have portrayed an ability to discern the intended expression of ragas at above chance level [6]. As to the reason why these four emotions were selected, there is some empirical evidence suggesting that these are among the most culturally common facial expressions of emotion identified between groups [7]. It is even possible to recognize musical affect expressions in performance across cultures – though it is easier to comprehend them within the same culture [8]. The emotion rating scale was presented to the participants

in the form of images taken from the Montreal set of facial displays of emotion [9], and then graded and morphed based on the *Facial Action Coding System* [10].

Acknowledging that there is an on-going debate on the variability of emotion in facial expressions (see [11, 12], we opted to utilise the Montreal set of facial displays of emotion, due to the fact that it offers a wide variety of encoders from different cultural backgrounds. As part of the pre-assessment tests, participants in NW Pakistan were presented with all female encoders (16) belonging to four different cultural groups, and were asked to select which encoder best represented a member of their group. Although during the pilot test we had an indication as to which possible encoders may match the participants' choice as to who may be viewed as a member of the Kalash and Khow tribes (encoders 25 and 27), we opted for repeating this stage during the experiment proper in order to ensure that the participants would select an encoder that they were comfortable with. Participants in Pakistan were presented with the two self-report tools as A4 prints on top of magnetic sheets, and indicated their choices by placing small physical markers on top of the prints. The physical markers were magnetic Ludo chips; they were selected due to the fact that Ludo (and its predecessor Pachisi) are very popular games in northwest Pakistan, thus setting the participants further at ease with the presentation and rating method.

References

1. Russell JA, Barrett LF. Core affect, prototypical emotional episodes, and other things called emotion: Dissecting the elephant. Journal of Personality and Social Psychology. 1999;76(5):805–819.

2. Cespedes-Guevara J, Eerola T. Music communicates affects, not basic emotions – A constructionist account of attribution of emotional meanings to music. Frontiers in Psychology. 2018;9:215. doi:10.3389/fpsyg.2018.00215.

3. Bradley MM, Lang PJ. Measuring emotion: The self-assessment manikin and the semantic differential. Journal of Behavior Therapy and Experimental Psychiatry. 1994;25(1):49–59.

4. Morris JD. Observations: SAM: the Self-Assessment Manikin; an efficient cross-cultural measurement of emotional response. Journal of Advertising Research. 1995;35(6):63–68.

5. Becker J. Deep Listeners: Music. Indiana University Press; 2004.

6. Balkwill LL, Thompson WF. A cross-cultural investigation of the perception of emotion in music: Psychophysical and cultural cues. Music Perception. 1999;17(1):43–64.

7. Jack RE, Sun W, Delis I, Garrod OG, Schyns PG. Four not six: Revealing culturally common facial expressions of emotion. Journal of Experimental Psychology: General. 2016;145(6):708.

8. Laukka P, Eerola T, Thingujam NS, Yamasaki T, Beller G. Universal and culture-specific factors in the recognition and performance of musical emotions. Emotion. 2013;13(3):434–449.

9. Beaupré M, Cheung N, Hess U. The Montreal set of facial displays of emotion. Montreal, Quebec, Canada; 2000.

10. Ekman P, Friesen WV. Facial action coding systems. Consulting Psychologists Press; 1978.

11. Jack RE, Garrod OG, Yu H, Caldara R, Schyns PG. Facial expressions of emotion are not culturally universal. Proceedings of the National Academy of Sciences. 2012;109(19):7241–7244.

12. Sauter DA, Eisner F. Commonalities outweigh differences in the communication of emotions across human cultures. Proceedings of the National Academy of Sciences. 2013;110(3):E180–E180.
