# Supplementary material for: Harmonic organisation conveys both universal and culture-specific cues for emotional expression in music
Source: PLoS One. 2021 Jan 13;16(1):e0244964. doi: 10.1371/journal.pone.0244964 (PMC7806179; doi:10.1371/journal.pone.0244964)
Supplement: S3 Appendix — A summary of the data analysis operations carried out before the main analysis operations including the consistency analysis of all ratings. (PDF) [file pone.0244964.s003.pdf]

S3 Appendix

Data analysis

The rating scales and measures were identical across the three groups with one exception, where the Pakistan groups rated emotions using the facial expressions of the Montreal Set of Facial Displays of Emotion (MSFDE), which allowed the participants choose the most likely emotion (anger, fear, sadness, happiness) and also intensity (0, 25, 50, 75, 100). These participants could nominate several emotions, although this happened only in the minority of cases. To make the ratings comparable to each other, the categories were converted into Likert scales representing each of the four emotions by assigning the intensity to the targeted emotion and converting the response to 1-5 (e.g., if a participant chose only happiness and intensity of 50, this would be converted into happiness 3 whereas the other emotions receive the rating of 1, the minimal rating).

Prior the main analysis, the consistencies of the responses of were examined using intra-class coefficients (Cronbach's α) for ratings of dimensions and emotions across groups and experiments (see Table 1). For speech, the inter-rater similarities are high (> 0.89, the most are > 0.96) and for music, the consistency is even higher (> 0.93) with one notable exception of Anger ratings for Kalash participants ($\alpha = 0.783$). For the artificial stimuli (melodies with different harmonisations), ratings of basic emotions for Khalash and Khow participants provided three noteworthy inconsistencies, namely for Happiness, where the assessments by Kho ($\alpha = 0.506$) participants were internally inconsistent. More curiously, the ratings of sadness for all stimuli were diverse and inconsistent among Kho ($\alpha = -0.256$) participants, and the same group scored relatively low consistency for Anger ratings as well ($\alpha = 0.644$). Since the ratings of both northwest Pakistan groups for the three dimensions were consistent and the same participants also made consistent ratings of basic emotions for speech and real music stimuli, the likely reason for the inconsistencies seem to relate to the artificial stimuli itself. For all three inconsistently rated emotions, the harmonisation stimuli elicited very low ratings in these basic emotions (see S4 Fig for details, the median rating for Sadness, Happiness, and Anger for Kho participants was 1 for each emotion). In the visual rating system the Kho participants used (MSFDE), they first chose the emotion category and then the intensity, and therefore the median rating of 1 suggests that for the majority of the stimuli they did not select any of these categories (see conversion example above). In case that one of these emotions were chosen, the intensities were low and participants often chose different basic emotions, leading to a widely different pattern of responses in terms of the scales. The fact that these basic emotion ratings essentially flatlined for harmonisations is nevertheless an interesting observation that warrants further research.

All the data was analysed with *generalized linear mixed models* [1–3] utilising the raw ratings.

References

1. R Core Team. R: A Language and Environment for Statistical Computing; 2017. Available from: `https://www.R-project.org/`.

Table 1. Consistency (α) across experiments and participant groups.

Scale	Speech			Music			Harmonisations		
	UK	Kho	Kalash	UK	Kho	Kalash	UK	Kho	Kalash
Happiness	0.991	0.993	0.995	0.968	0.975	0.961	0.973	0.506	0.800
Sadness	0.982	0.992	0.995	0.985	0.958	0.945	0.949	-0.256	0.893
Anger	0.986	0.994	0.988	0.989	0.943	0.783	0.974	0.644	0.788
Valence	0.894	0.982	0.992	0.932	0.958	0.977	0.956	0.728	0.950
Energy	0.939	0.962	0.983	0.977	0.974	0.988	0.874	0.794	0.950
Dominance	0.968	0.963	0.968	0.965	0.936	0.971	0.888	0.846	0.968

2. Bates D, Mächler M, Bolker B, Walker S. Fitting linear mixed-effects models using lme4. Journal of Statistical Software. 2015;67(1):1–48. doi:10.18637/jss.v067.i01.

3. Revelle W. psych: Procedures for Psychological, Psychometric, and Personality Research; 2019. Available from: `https://CRAN.R-project.org/package=psych`.
