# Supplementary material for: Harmonic organisation conveys both universal and culture-specific cues for emotional expression in music
Source: PLoS One. 2021 Jan 13;16(1):e0244964. doi: 10.1371/journal.pone.0244964 (PMC7806179; doi:10.1371/journal.pone.0244964)
Supplement: S4 Appendix — A summary of ethical considerations before entering the field, including a concise briefing as to the status, role and presence of music among the two tribes in northwest Pakistan. (PDF) [file pone.0244964.s004.pdf]

S4 Appendix

Practical considerations and additional information regarding the Kalash and Khow musical practices.

Ethical considerations

Through the presence and compliance of local community paragons and the government of Pakistan, the researchers ensured that the implementation of the study would not affect the non-Western participants in any adverse way, and would not disrupt the local way of life and culture during the researchers' physical presence and after their leave. The research project introduced the idea that music may include emotional connotations, and that listeners may identify said emotions expressed via music. As both communities share the belief that this is indeed possible, and evident through their own performance culture, there was no peril that the research experience would be uncomfortable by introducing a novel idea. This was further mitigated through the research design, which did not involve telling participants that there is a right or wrong response in rating the emotional content of the speech/music presented to them; this would be counter-productive to the research goals themselves. Part of this consideration was to ensure that participants were fully aware of the goals and aspirations of the project; and that the research was carried out with respect towards participating individuals and members of the community, and in accordance with the hosting institution's ethics in research guidelines.

The majority of the Kalash and Kho participants originated from Bumburet and Rumbur valley in the district of Chitral, northwest Pakistan. Pakistani schooling in the Khyber-Pahtunkwa region does not feature elements of Western music education; therefore it is natural that Kalash and Kho musicians would not display any familiarity with Western musical styles or exposure to any notational system. Though formal music training is often used as a way to determine whether someone possesses musical ability, this method of separation would be inappropriate among the Kalash and Kho participants – a point also mentioned by [1].

Additional information regarding local musical practise among the Khow and Kalash tribes

Music among the Kalash and the Kho is a highly communal activity. Active performers are not separated from a non-participating audience who are actively involved in the performances themselves (mainly among the Kalash), though there are individuals who are celebrated for their performing skills among both communities. Younger people in particular often express the will to perform and record their own compositions, either during evening gatherings were music is played, or, provided that they have the budget available, record their music at a local recording studio in Chitral (which is about 2 hours away) and then share their songs with their friends and family. Music instrumentalists and vocalists alike are not literate in any form of music notation; music is usually taught either by direct imitation and tutelage with the assistance of an older mentor, relative or peer, or by self-practicing with audio cassette recordings of local well-known performers, though this is not very common.

Both Kalash and Khow communities have retained their own unique traditional musical cultures, as these are central to their identity and social life in their region. The

entire region of Chitral and broader Khyber Pakhtunkhwa is unique, in that "unlike many other Muslim societies in which... music, conversation and food have diminished because of the expansion of mass-mediated music, television, and film" [2, p. 75], people from both communities continue to organise and attend evening social events, where traditional music and dancing take place.

It is important to point out that performance culture among the Kho is based on the outward expression of emotion through music. Music and dance gatherings take place on joyous occasions (istoks) and during evening mahfils (lit. "polite gatherings"). Istoks take place during weddings, athletic victories of local cricket and polo teams, or any non-ritual joyous gathering of friends (for a detailed description see [3]. The music is based on highly energetic fast-paced drumming on two drums (dol and dumduma) often accompanied by two or more high-pitched double-reed instruments (surnai) or a flute (belu) performing at their maximum loudness capability and accompanied by fervent ululations and singing with the purpose of expressing happiness. In the evening mahfils, poetry and songs which express emotions such as melancholy, longing, sadness and (unfulfilled) love, are performed by a trio of musicians playing a chitrali setar, a drum (gilliken), and a vocalist. In terms of tempo, songs usually range from slow (dani) to mid-tempo (sauz) speed, whereas loudness levels remain relatively low as these are indoor all-male gatherings (usually) in patrons' living rooms. Musicians and participating audience alike "actively reflect upon their conceptions and experiences of emotions which they themselves are experiencing, and recognise as present in the music" [4, p. 92].

At the same time, the Kalash, though influenced by their neighbouring tribes in their non-ritual music, zealously adhere to their own ritual music and customs. The manifestation of both happiness, sadness, as well as power/praise, make themselves prominent in fervent outward expressions of emotion during social events, such as weddings, funerals, and through panegyric ritual music in festivals such as the Joshi, the Ucaw and the Chawmos [5]. For the Chawmos festival, there is even a wide repertory of flirtatious, courting and even lewd songs expressing a wide array of complex yet positively valenced emotions [6, 7]. Kalash ritual dancing acquires different interpretations based on the style and the occasion: in terms of tempo, the three most common dances are either slow (drasailak), mid-tempo (dushak) or fast-paced (ca), and are performed with the accompaniment of two drums: a dau, a bass drum which is similar to the doll, and a wac which is an hourglass-shaped drum, together with female harmonic chanting in drone chromatic clusters and male (honourific) singing. In terms of melancholy, sadness, unrequited love, and joy, the Kalash express these emotions through performance gatherings similar to the mahfil (as presented for the Kho) but in a usually outdoor setting of mixed company, and with a different line-up which includes more percussionists and a flute (ispnoe) instead of the Chitrali setar.

Neither Kalash nor Khow music is organised according to mode distinction (major/minor), but rather on performance situation and style; data have been collected regarding the recognition and the awareness for these pitch structural differences and will be presented at a later stage (current authors, in preparation). Khowari music has immense richness in terms of performance style, and significant depth and variation in terms of melodic modes and tempos used during different performance settings and music (dani and non-dani alike). At the same time, Kalash non-ritual music is highly imitative of local performance styles from their immediate neighbouring tribes (Kho, Pashtuns and Nuristani, to name but a few), and, as the majority of peoples in the region are multi-lingual, it is not uncommon to hear songs from one community being sung by musicians of another community in one of their evening performance sessions. Kalash ritual music is, however, unique to the Kalash, and it is used as a cultural identifier. None of the neighbouring tribes comprehend its performance settings, values

or organisation, and nor do they wish to do so, as direct association with the Kalash cultural practices is not considered appropriate due to religious differences. Regarding melodic context, while Kalash (ritual) music incorporates high levels of roughness and dissonance (see next paragraph), not all of their music is as such; during the winter festival (Chawmos) Kalash songs are either major or minor pentatonic, with the difference in mode between major and minor is, as already stated,irrelevant to the connotation of the songs whose lyrics are usually flirtatious, courting and even lewd in content.

In terms of harmonisation, the concept of Western tonal (vertical) alignment is not the prevalent norm in both tribes and in the entire region as well. It has to be pointed out that not all Kalash music is characterised by roughness and dissonance; rather, it is present in the communities under discussion either in the form of heterophony between the leading voice and accompanying instruments (most commonly the Chitrali setar or the flute), through parallel harmonisation sung as chromatic cluster drones during ritual music, and melody-dominated homophony which is present not only in traditional music during evening song performances, but in contemporary music styles as well. Full chordal accompaniment for both communities is known through Urdu pop music and local modern compositions which, though listened to when available, does not appear to influence local performance practices, but rather enriches them (for example, contemporary Khow mahfils may incorporate more modern songs as well, but not Urdu pop music which is considered a different style altogether). Bollywood films and Punjabi/Hindi music are non-existent and strongly frowned upon, particularly by the Kho. The overwhelming majority do not have access to radio or to a television set, with the majority of people investing on mobile phones instead. From our Kho participants, 41.17% had access to a radio, and 35.3% had access to a TV set. From the Kalash participants, 17.65% had access to a radio, whilst a further 8.82% had access to a TV set. However, these numbers have to be put into appropriate context: when participants refer that they listen to the radio, they mean the one available on a car, which they listen to on their way to and from Chitral, and only whenever the reception is good or any of the available radio stations actually play music (local Khowari music, or on occasion, Urdu pop) instead of the news, or recitations of holy scripture. A such, music is listened through mobile phones where songs are shared through Bluetooth connection. Keeping a mobile phone charged with power, and recharging it at any and every given opportunity, is on top of everyone's list. Owning such items are more of a status symbol of affluence, and something to be prominently displayed inside the household, despite the fact that the majority of electrical devices are in effect of very little use; there's extremely poor signal reception, electricity is only available at night, and it can provide stable power of roughly 200W per house. Anything more demanding than this in terms of power (as for example, a hair dryer, a water kettle or a clothes iron), would cause irreparable damage to the power grid of a household with members daring enough to put such a device into a power socket, as there are no electricity safety boxes. Nevertheless, this is enough to power a small television set if all other devices (incl. mobile phone chargers are unplugged), especially if they are of a newer type with LED backing lights. Neither radio nor television sets (at least as far as those participants who professed to having access to one) have access to Western music or culture from these media, either due to poor reception of lack of accessibility.

References

1. Jacoby N, Margulis EH, Clayton M, Hannon E, Honing H, Iversen J, et al. Cross-cultural work in music cognition. Music Perception. 2020;37(3):185–195.

2. Shannon JH. Emotion, performance, and temporality in Arab music: Reflections on Tarab. Cultural Anthropology. 2003;18(1):72–98.

3. Marsden M. All-male sonic gatherings, Islamic reform, and masculinity in northern Pakistan. American Ethnologist. 2007;34(3):473–490.

4. Marsden M. Love and elopement in northern Pakistan. Journal of the Royal Anthropological Institute. 2007;13(1):91–108.

5. Parkes P. Personal and collective identity in Kalasha song performance: The significance of music-making in a minority enclave. In: Stokes M, editor. Ethnicity, identity, and music: the musical construction of place. Ethnicity and identity series. Oxford, UK: Oxford; 1994. p. 157–187.

6. Cacopardo AS. The winter solstice festival of the Kalasha of Birir: Some comparative suggestions. Acta Orientalia. 2008;69:77–121.

7. Cacopardo AS. Pagan Christmas: Winter Feasts of the Kalasha of the Hindu Kush. Gingko Library; 2017.
