# Supplementary material for: Harmonic organisation conveys both universal and culture-specific cues for emotional expression in music
Source: PLoS One. 2021 Jan 13;16(1):e0244964. doi: 10.1371/journal.pone.0244964 (PMC7806179; doi:10.1371/journal.pone.0244964)
Supplement: S5 Appendix — A report on how the harmonisation stimuli were analysed in terms of basic emotions. (PDF) [file pone.0244964.s005.pdf]

S5 Appendix

Additional analyses

Basic emotion ratings

In the main analyses, we focus on three dimensions since many of the basic emotion ratings correlate with the dimensions and some of the basic emotions received low overall ratings (e.g., fear) and were inconsistently rated between the participants (Kho and Khalash, see *SI Data Analysis*). However, a similar analysis operations was carried out for the three basic emotions (Anger, Sadness, and Happiness) as was reported for the dimensions.

None of the harmonisations utilised is able to convey emotional expressions in terms of basic emotions in a clear and strong fashion (it should be pointed out that the stimuli were identical in terms of volume, tempo, timbre, attack rate, and expressivity), but there are nevertheless interesting cultural differences. Firstly, the ratings of Anger do not exhibit any significant main effect of Culture (t(3257,169)=-0.31, p=.76) but Harmonisation style (t=14.0, p<.001) and strong interaction (t=-8.09, p<.001). For anger, the Whole-tone harmonisation style conveys this expression to some degree to UK participants (M=2.82) but not for Kalash (M=1.86, t=-6.91, p<.001) or Kho participants (M=1.62, t=-8.65, p<.001). For happiness, the harmonisation style in major key (Jazz Major) are rated as expressing a higher degree of happiness by the UK participants (M=1.52) in comparison to non-Western participants (Jazz major for Kho, M=1.00, t=-2.948, p<.001, and Kalash, M=1.10, t=-3.70, p<.001) although this relationship does not hold for Bach major excerpts. The rest of the conditions elicit minor differences but since these excerpts express happiness only weakly, we refrain from drawing attention to these. Finally, ratings of sadness exhibit the main effect of Culture (t(3257, 169)=-5.22, p<.001) and Harmonisation style (t=3.43, p<.001) but no interaction (t=-0.84, p=40). Interestingly, the solo version of these melodies as well as the Organum harmonisations operate in a similar fashion where the UK participants consider these to express sadness at a higher degree (M=2.76 and 2.85, respectively) than the other groups (M=1.96 and 2.42 for Kalash, and M=1.64 and 1.79 for Kho, all significantly lower than the UK ratings, p<.05). However, the Kalash harmonisation style displays the largest discrepancy between the three groups, where the Kalash participants attribute sadness as the expression conveyed (M=3.54) whereas the Kho participants do not hear sadness being expressed at all (M=1.71, t=8.57,p<.001).

Nevertheless, the sadness ratings bring out certain noteworthy cultural differences; the harmonisations using the minor mode (Bach Minor, Jazz Minor) received significantly higher ratings from the UK participants (Bach minor, M=2.88, Jazz minor M=2.71) than from the Kalash (Bach minor, M=2.26, t=8.82, p<.001, or Jazz minor, M=2.26, t=2.52, p<.05) or Kho (Bach minor, M=1.85, t=5.78, p<.001, or Jazz minor, M=1.66, t=5.94, p<.001). This is in line with our hypothesis that cultural competence is needed to decode the emotional connotations of the major/minor distinction.

S7 Fig. Participants' ratings for the different harmonisation styles in terms of Anger, Happiness and Sadness.

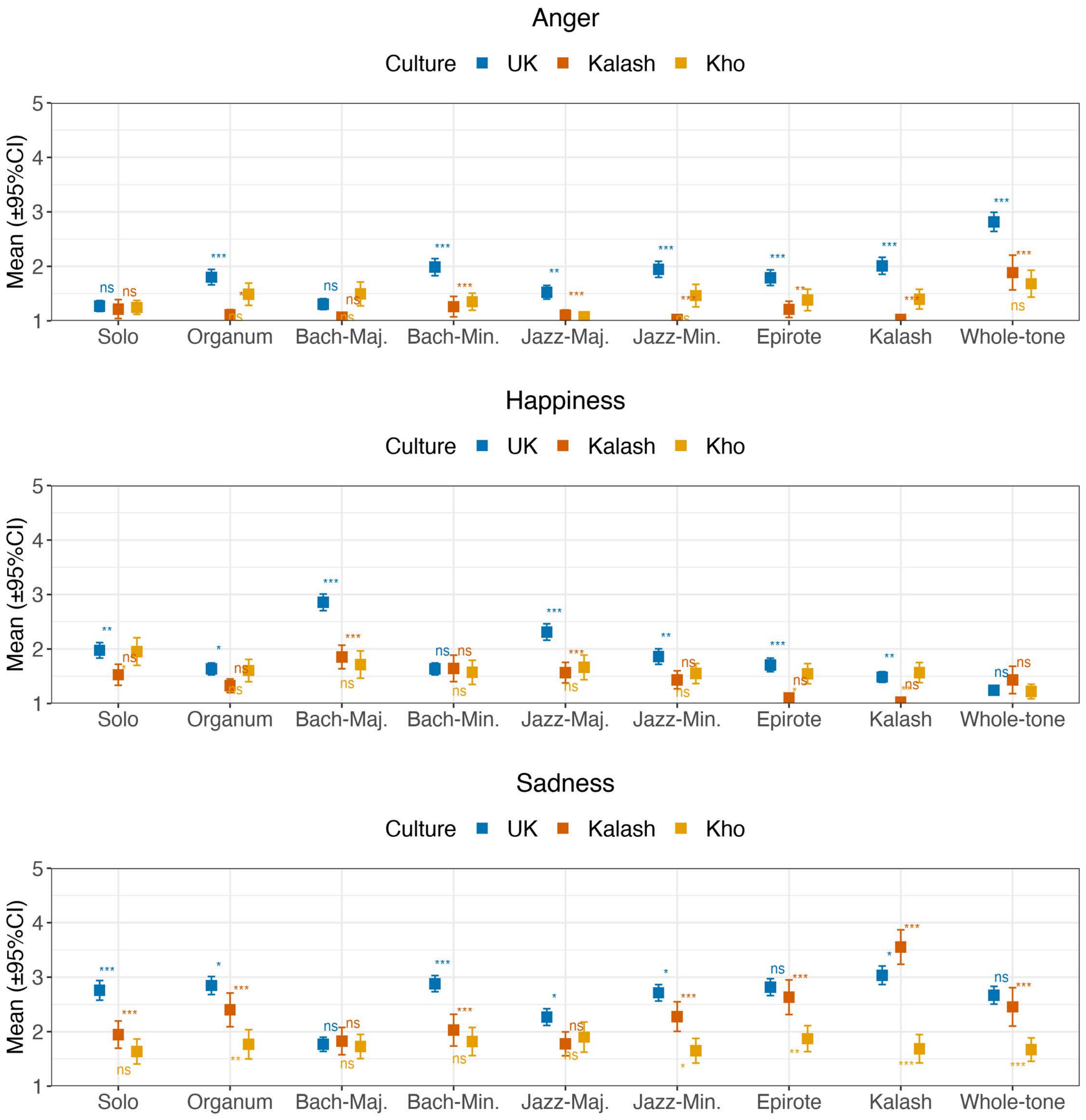
Anger
Culture UK Kalash Kho
Mean (±95%CI)
Solo
Organum
Bach-Maj.
Bach-Min.
Jazz-Maj.
Jazz-Min.
Epirote
Kalash
Whole-tone
Happiness
Culture UK Kalash Kho
Mean (±95%CI)
Solo
Organum
Bach-Maj.
Bach-Min.
Jazz-Maj.
Jazz-Min.
Epirote
Kalash
Whole-tone
Sadness
Culture UK Kalash Kho
Mean (±95%CI)
Solo
Organum
Bach-Maj.
Bach-Min.
Jazz-Maj.
Jazz-Min.
Epirote
Kalash
Whole-tone
